# PU-GRAIL: residue-level graph learning for identifying protective bacterial antigens under positive-unlabeled supervision

**DOI:** 10.1093/bioinformatics/btag263

**Published:** 2026-07-07

**Authors:** Jaemin Jeon, Sangwook Jung, Inuk Jung, Kwangsoo Kim, Jinki Yeom

**Affiliations:** Interdisciplinary Program in Bioinformatics, Seoul National University, Seoul 08826, Republic of Korea; Department of Medicine, Seoul National University, Seoul 03080, Republic of Korea; School of Computer Science and Engineering, Kyungpook National University, Daegu 41566, Republic of Korea; Department of Medicine, Seoul National University, Seoul 03080, Republic of Korea; Department of Transdisciplinary Medicine, Seoul National University Hospital, Seoul 03080, Republic of Korea; Center for Data Science, Healthcare AI Research Institute, Seoul National University Hospital, Seoul 03080, Republic of Korea; Interdisciplinary Program in Bioinformatics, Seoul National University, Seoul 08826, Republic of Korea; Department of Biomedical Science, College of Medicine, Seoul National University, Seoul 08826, Republic of Korea; Department of Microbiology and Immunology, Seoul National University, Seoul 08826, Republic of Korea; Cancer Research Institute, Seoul National University, Seoul 03080, Republic of Korea

## Abstract

**Motivation:**

The identification of protective antigens is fundamentally constrained by sparse annotations and pervasive label uncertainty in reverse vaccinology. Protective antigen–antibody interactions are mediated by a limited subset of surface-accessible residues that form spatially coherent epitopes. This motivates modeling antigenicity at the residue level, where 3D structure provides critical context for functional immune recognition. Moreover, antigen datasets are inherently positive unlabeled: unannotated proteins may contain hidden positives, making reliable negatives difficult to obtain.

**Results:**

We present PU-GRAIL, a graph neural network framework that integrates protein language model embeddings with predicted 3D structures under positive-unlabeled learning. Trained and evaluated on three benchmark datasets, PU-GRAIL achieves competitive performance compared with existing methods. Importantly, the model’s attention mechanism enables residue-level interpretation, identifying putative epitope regions that correspond to experimentally validated antibody-binding sites. Beyond standard benchmarks, we demonstrate practical utility through (i) severity-associated antigenicity patterns in SARS-CoV-2 patient cohorts, and (ii) proteome-wide vaccine candidate prioritization across 11 bacterial species.

**Availability and implementation:**

The PU-GRAIL software is available at https://github.com/jaeminjj/PU-GRAIL.

## 1 Introduction

Infectious diseases remain a leading cause of morbidity and mortality worldwide, and vaccination represents one of the most effective public health interventions to reduce this burden (Burton *et al.* 2009). Accordingly, vaccine development critically depends on identifying *protective antigens*—proteins that elicit immune responses sufficient to protect against infection or disease. In general, protective antigens induce neutralizing antibodies, T-cell responses and support durable immunological memory, enabling rapid protection upon subsequent pathogen encounters (Pulendran and Ahmed 2006). Experimentally screening entire pathogen proteomes for protective antigens is prohibitively time-consuming and costly. Reverse vaccinology (RV), first applied to the development of a *Neisseria meningitidis* serogroup B vaccine (Masignani *et al.* 2002), addresses this challenge by computationally prioritizing candidate antigens for experimental validation. By narrowing the experimental search space, RV reduces development time, cost, and labor while enabling systematic exploration of proteins that are difficult to identify using conventional approaches.

A variety of computational tools have been developed to support RV pipelines. Early approaches focused on sequence-based features such as subcellular localization, signal peptides, transmembrane topology, and adhesin-like properties to filter surface-exposed or secreted proteins (Vivona *et al.* 2006). Machine learning methods subsequently integrated these biological features with evolutionary conservation and physicochemical properties to improve performance. Representative examples include Vaxign-ML (Ong *et al.* 2020), VaxiJen2.0 (Doytchinova and Flower 2007), and VaxiJen3.0 (Dimitrov *et al.* 2020), which use ensemble learning on curated feature sets derived from protein sequences. VirusImmu (Li *et al.* 2025) represents a specialized approach trained specifically on viral protein data, though its applicability to bacterial protective antigens remains limited. The representation of protein sequences has evolved considerably with the advent of protein language models. Traditional methods relied solely on handcrafted biological features, whereas modern approaches leverage pretrained embeddings from transformer-based models such as ESM (Rives *et al.* 2021) or ProtBERT (Elnaggar *et al.* 2022). PAPreC (Martins *et al.* 2025) exemplifies this paradigm by utilizing ESM embeddings for protective antigen prediction. Deep learning architectures have further advanced the field; Vaxi-DL (Rawal *et al.* 2022) uses neural networks trained on sequence representations to classify antigenic proteins. Beyond antigenicity prediction, sequence embeddings have also been applied to predict clinical outcomes such as disease severity, demonstrating the versatility of these representations (Sidhom *et al.* 2021, Jeon *et al.* 2025). However, protective antigenicity is not solely determined by sequence composition; the 3D structure of a protein governs epitope accessibility, conformational epitope formation, and antibody binding affinity (Lo *et al.* 2013). The recent availability of high-accuracy protein structure prediction methods, including AlphaFold2 (Jumper *et al.* 2021), OmegaFold (Wu *et al.* 2022), and ESMFold (Lin *et al.* 2023), has made it feasible to obtain structural models for virtually any protein sequence. VenusVaccine (Li *et al.* 2024) represents a pioneering effort to integrate predicted structural information with sequence features for antigen prediction.

Despite these advances, opportunities remain for further improvement. Most existing approaches assume that unannotated proteins are non-protective, yet in practice many unlabeled sequences may include undiscovered antigens, suggesting that positive-unlabeled learning strategies could improve robustness. Additionally, antibody–antigen recognition is mediated by direct contacts between antibodies and a limited subset of surface-accessible residues, implying that explicitly modeling these residue-level interactions could provide more biologically meaningful signals for identifying protective antigens.

To address these challenges, we present PU-GRAIL, a graph neural network framework that integrates protein language model embeddings with predicted 3D structures under a positive-unlabeled (PU) learning paradigm. The model constructs residue-level graphs that separately encode sequential and structural connectivity, and uses attention-based pooling to identify putative epitope regions. Crucially, this attention mechanism provides residue-level interpretability: high-attention residues mapped onto experimentally resolved antibody-antigen complexes localize within 4.5 Å of antibody residues demonstrating that the model learns biologically meaningful epitope features without explicit structural supervision. Extensive experiments across benchmark datasets spanning bacteria, viruses, and tumors demonstrate that PU-GRAIL achieves competitive performance compared to existing methods. We further demonstrate its practical utility through severity-associated antigenicity analysis in SARS-CoV-2 and candidate prioritization across bacterial proteomes.

**Table 1:** Summary statistics of the benchmark datasets, including the number of positive and negative samples, mean sequence length (in amino acids), and the proportion of protective sequences.

Dataset	Positive	Negative	Mean length	Ratio (%)
Bcipep	1011	204	16.5	91
HLA	1032	5727	17.2	15
ImmunoDB-bacteria	913	1562	348.9	37
ImmunoDB-virus	2078	1886	389.5	53
ImmunoDB-tumor	300	777	253.3	38
Gram+ Protegen	57	40	381.2	59
Gram– Protegen	75	72	377.8	51
All Gram protein	2000	2000	253.8	50
All Gram epitope	2000	2000	15.7	50

## 2 Datasets

To ensure a fair and comprehensive comparison with previously published antigenicity prediction models, PU-GRAIL was evaluated on the same benchmark datasets (Table 1) .

ImmunoDB dataset: This dataset was originally established in the VenusVaccine study as a comprehensive benchmark for antigenicity prediction. It integrates experimentally validated immunogenic and non-immunogenic protein sequences across three biological domains (bacteria, viruses, and human tumor associated proteins) providing a unified resource for assessing antigenic potential across diverse organisms. Positive samples were curated from the Protegen (Yang *et al.* 2011) database, the Immune Epitope Database [IEDB (Vita *et al.* 2019)], and peer-reviewed literature, and include proteins that have been experimentally verified to elicit protective immune responses in humans or relevant animal models. Negative samples were obtained from complete proteomes downloaded from UniProtKB (Pundir *et al.* 2017), filtered to ensure less than 30% sequence identity with any positive sequence using BLAST, and further screened with VaxiJen to retain only entries predicted as Probable Non-Antigen.

PAPreCdatasets: To evaluate generalization across antigenic resolutions and bacterial groups, PU-GRAIL was evaluated on benchmark datasets from the PAPreC framework, including Bcipep (Saha *et al.* 2005), HLA (Dhanda *et al.* 2018), all gram epitope, and all gram protein datasets from Protegen. These datasets cover both epitope level and protein level antigenicity and include Gram-positive and Gram-negative bacterial species. Bcipep and HLA consist of experimentally validated B-cell and T-cell epitopes curated from the Immune Epitope Database (IEDB) and related repositories. The protein-level datasets were constructed from positive protective antigens in the Protegen database and negative samples generated from complete bacterial proteomes in UniProtKB, with negatives selected to have <30% sequence identity to positive antigens using BLAST.

Vaxign-ML datasets: This dataset was used as an independent test benchmark to evaluate model performance across different bacterial groups, following the Gram-specific antigenicity setting introduced in Vaxign-ML. The benchmark consists of experimentally validated protective antigens curated from the Protegen database, paired with non-antigens sampled from complete bacterial proteomes. The independent test dataset provided by Vaxign-ML was used exclusively for external evaluation, while the Protegen dataset was used for model training. To ensure strict data independence, any sequences overlapping between the Protegen training set and the Vaxign-ML test dataset were removed prior to evaluation. The trained model was then evaluated on the Gram-positive and Gram-negative test sets defined in Vaxign-ML.

Model performance was assessed using 5-fold cross-validation, where each fold preserved the original class distribution of the dataset. In each fold, data were split into training, validation, and test sets, ensuring that no sequence instance appeared in more than one split. All hyperparameters were selected based on validation performance, and final results were reported on the independent test sets. Train-test overlap was validated across all folds and results are provided in Table 1, available as supplementary data at *Bioinformatics* online.

## 3 Materials and methods

**Figure 1:**
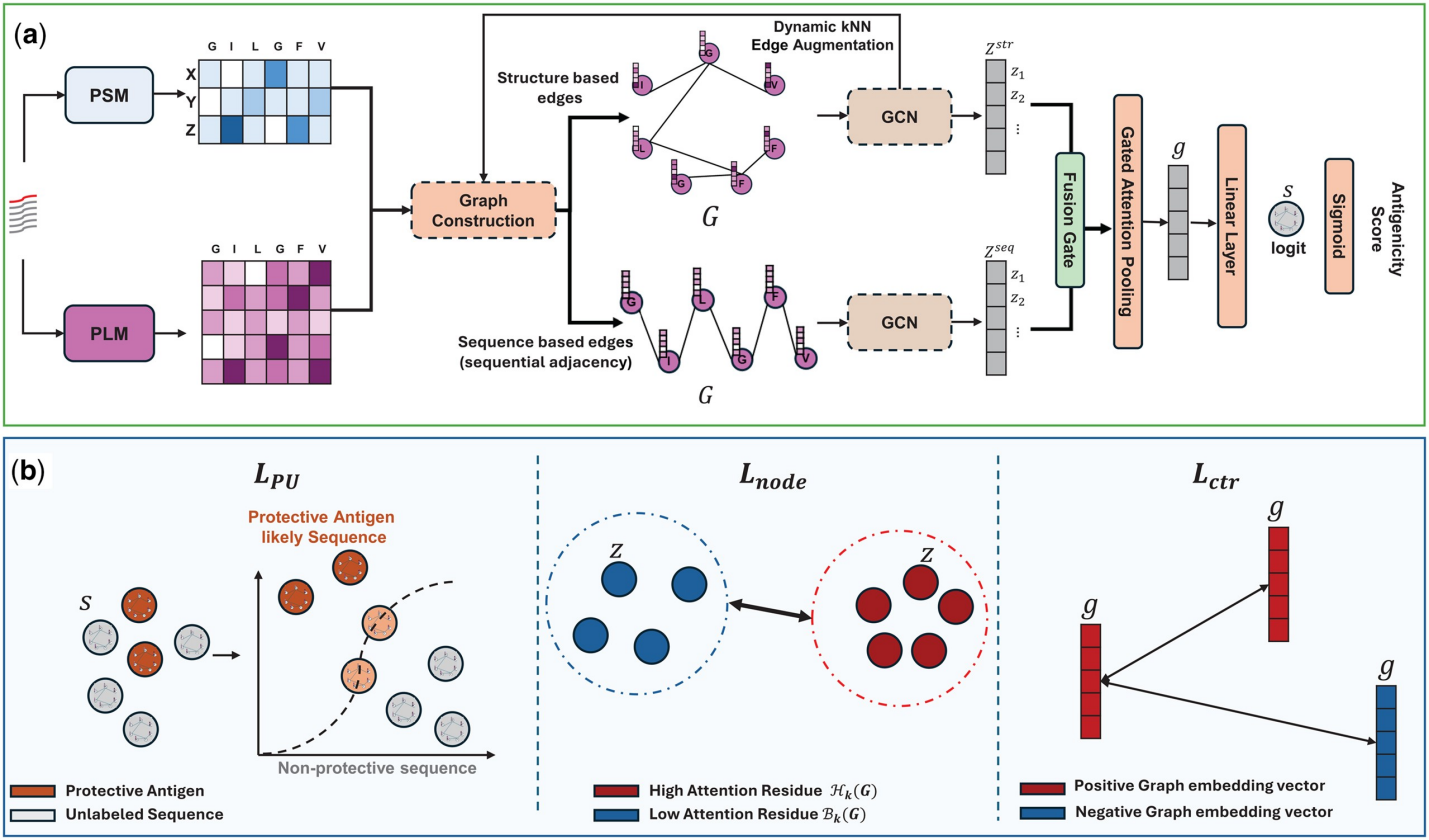
(a) Residue-level graph construction and antigenicity scoring with dynamic kNN edge augmentation, fusion gating, and gated-attention pooling. (b) Training objectives: PU ranking loss ($L_{PU}$), node-level separation loss ($L_{\mathrm{node}}$) using high-/low-attention residue sets H(g) and B(g), and graph-level contrastive loss ($L_{\mathrm{ctr}}$).

PU-GRAIL predicts protective antigenicity of a protein under a positive-unlabeled (PU) learning framework by combining a protein structure model (PSM) and protein language model (PLM) in Fig. 1.

### 3.1 Graph construction

Given a protein sequence of length *L*, we represent it as a weighted residue graph G=(N,E), where $N={\left\{n_{i}\right\}}_{i=1}^{L}$ denotes residue nodes and each $n_{i}$ corresponds to the *i*th residue. The edge set E specifies residue–residue connections used for message passing.

Specifically, PU-GRAIL uses ESM-2 to obtain per-residue embeddings of dimension 1280. We project these to 256 dimensions via a two-layer MLP, then concatenate with 20D one-hot amino-acid encoding, yielding a 308D node feature vector for GNN input. The GCN produces residue embeddings of dimension 128. Using a protein structure model (ESMFold), we obtain predicted C${}_{\alpha }$ coordinates $r_{i}\in R^{3}$ for each residue. We define structure-driven connectivity by connecting residue pairs within a distance threshold τ:


$$(i,j)\in E_{\text{str}}\;\text{if}\;||r_{i}-r_{j}|{|}_{2}\leq \tau ,$$

where τ is set to 8 Å. To preserve backbone continuity, we also include sequential edges between adjacent residues:


$$(i,i+1)\in E_{\text{seq}},\;(i+1,i)\in E_{\text{seq}},\;E=E_{\text{str}}\cup E_{\text{seq}}.$$

For each structural edge $(i,j)\in E_{\text{str}}$, we assign a distance-based weight


$$w_{ij}^{\text{str}}=\text{exp}\;\left(-\frac{||r_{i}-r_{j}|{|}_{2}^{2}}{2\sigma^{2}}\right),$$

while sequential edges use a constant weight. These edge weights are used for weighted message passing in the GCN encoder.

### 3.2 Dynamic graph refinement for antigenicity scoring

Given the weighted residue graph G=(N,E) with |N|=L residues, we apply a stack of two GCN layers (hidden dimension 128) to propagate information across residues and obtain structure-aware residue embeddings ${\left\{z_{i}^{\text{str}}\right\}}_{i=1}^{L}$ and sequence derived residue embeddings ${\left\{z_{i}^{\text{seq}}\right\}}_{i=1}^{L}$.

To capture functional similarity beyond geometric proximity, we perform dynamic *k*-nearest-neighbor (kNN) edge augmentation during training. We compute cosine similarities in the current structural embedding space ${\left\{z_{i}^{\text{str}}\right\}}_{i=1}^{L}$ and add *k*-nearest-neighbor edges (k=24) between residues with highest cosine similarity. These similarity edges are merged with the original edge set E, and duplicated edges retain the stronger affinity (maximum weight). The augmented graph is then used in subsequent message passing steps.

We fuse the two residue embeddings at the node level:


(1) $$z_{i}=\alpha z_{i}^{\text{seq}}+(1-\alpha )z_{i}^{\text{str}},$$

where α∈(0,1) is a learnable scalar gate parameterized as α=σ(β). Stacking ${\left\{z_{i}\right\}}_{i=1}^{L}$ yields the fused residue embedding matrix $Z=[z_{1};\ldots ;z_{L}]\in R^{L\times d}$. To obtain a protein-level (graph-level) embedding, we apply *gated attention pooling* to Z. Specifically, a small gating network assigns an unnormalized importance score to each residue:


$$a_{i}=\text{MLP}(z_{i}),\;w_{i}=\text{softmax}(a_{i}).$$

The graph embedding is computed as the weighted sum


(2) $$g=\sum_{i=1}^{L}w_{i}z_{i}.$$

A prediction head then maps g to a logit


(3) s=MLP(g),

and the final protective antigenicity probability is obtained by


(4) p=σ(s).

### 3.3 Learning objective

PU-GRAIL is trained by jointly optimizing three complementary objectives that capture (i) sequence-level positive-unlabeled supervision, (ii) residue-level discriminability, and (iii) structural consistency. The overall training objective is defined as a weighted sum:


(5) $$L=L_{\text{PU}}+\lambda_{\text{node}}L_{\text{node}}+\lambda_{\text{ctr}}L_{\text{ctr}}.$$

**PU-AUC loss:** In this study, only a subset of proteins are experimentally validated protective antigens (positives), whereas the remaining proteins are unlabeled and may include undiscovered positives. To train without explicit negatives, we adopt a positive-unlabeled AUC (PU-AUC) objective, which encourages *rank separation* by assigning higher scores to labeled positives than to unlabeled samples. Concretely, PU-AUC maximizes the probability that a randomly chosen positive receives a higher score than a randomly chosen unlabeled example, i.e. $\mathrm{Pr}(s_{p}>s_{u})$, making it well-suited for PU learning when reliable negatives are unavailable.

Let $s_{i}$ denote the predicted logit for the *i*th protein (Eq. 3), and let $y_{i}\in \{1,0\}$ indicate whether protein *i* is a labeled positive ($y_{i}=1$) or an unlabeled sample ($y_{i}=0$). Within a mini-batch of size *B*, we denote labeled positives by P and unlabeled examples by U. For stability, we center logits as ${\tilde{s}}_{i}=s_{i}-\bar{s}$, where $\bar{s}=\frac{1}{B}\sum_{k=1}^{B}s_{k}$, and stop gradients through $\bar{s}$. For each positive-unlabeled pair (p,u), define $d_{pu}={\tilde{s}}_{p}-{\tilde{s}}_{u}$. Because the AUC indicator $I(d_{pu}>0)$ is non-differentiable, we optimize a smooth pairwise logistic (softplus) surrogate:


(6) $$\ell (d_{pu})=\text{log}\;\left(1+\text{exp}(-\gamma d_{pu})\right)=\text{softplus}(-\gamma d_{pu}),$$

where γ>0 is a temperature parameter controlling the sharpness of the ranking penalty. The PU-AUC objective is computed by averaging over positive-unlabeled pairs within the mini-batch:


(7) $$L_{\text{PU}}=\frac{1}{\left|P||U^{\prime }\right|}\sum_{p\in P}\sum_{u\in U^{\prime }}\ell ({\tilde{s}}_{p}-{\tilde{s}}_{u}),$$

where $u^{\prime }\subseteq u$ is an optional random subset for efficiency and implicit hard-negative mining.

**Node-level separation loss:** Biologically, molecular recognition is typically mediated by a limited set of contact residues (e.g. at antibody paratope–epitope interfaces), rather than uniformly across all residues. Motivated by this, PU-GRAIL regularizes the gated-attention mechanism to avoid overly diffuse weights and to promote residue-level discriminability. Specifically, we introduce a node-level separation loss guided by the attention weights ${\left\{w_{i}\right\}}_{i=1}^{L}$. For each protein graph *G*, residues are ranked by $w_{i}$, and we define $H_{k}(G)\subseteq \{1,\ldots ,L\}$ and $B_{k}(G)\subseteq \{1,\ldots ,L\}$ as the index sets of the top-*k* and bottom-*k* residues, respectively.

Let ${\left\{z_{i}\right\}}_{i=1}^{L}$ denote the fused residue embeddings obtained after dual-branch graph encoding. We compute the mean embeddings of the high-attention and low-attention residue groups as


(8) $$\mu_{H}(G)=\frac{1}{\left|H_{k}(G)\right|}\sum_{i\in H_{k}(G)}z_{i},\;\mu_{B}(G)=\frac{1}{\left|B_{k}(G)\right|}\sum_{i\in B_{k}(G)}z_{i}.$$

To encourage separation between informative and uninformative residues, we maximize the similarity among high-attention residues while reducing the correlation between the two groups (computed over positives in the mini-batch):


(9) $$L_{\text{node}}=\sum_{G\in P}\left[(1-S_{HH}(G)+\lambda \left|sim(\mu_{H}(G),\;\mu_{B}(G))\right|+\beta sim(\mu_{H}(G),\;\mu_{B}{\left(G)\right)}^{2}\right],$$

where P denotes the set of positive protein graphs in the current mini-batch, and (·,·) denotes cosine similarity. $S_{HH}(G)$ denotes the pairwise cosine similarity matrix among high-attention residue embeddings within graph G. The second term suppresses similarity between informative and uninformative residue groups, while the third term acts as an orthogonality regularizer encouraging disentangled residue-level representations.

This regularizer encourages residues receiving high attention weights to form a compact and discriminative cluster in the embedding space, while pushing low-attention residues away.

**Graph-level contrastive loss:** To further stabilize protein-level representations and encourage discriminative structure in the embedding space, we introduce a supervised contrastive regularization on the graph embeddings $\left\{g_{i}\right\}$, where each $g_{i}$ denotes the pooled representation of a protein sequence obtained by gated attention pooling (Eq. 2).

Under the positive-unlabeled (PU) setting, only positive proteins are reliably annotated. We therefore treat each positive example i∈P as an anchor and define P(i)=P∖{i} as the set of other positive samples within the same mini-batch. Given two graph embeddings $g_{i}$ and $g_{j}$, we measure their similarity using cosine similarity $\text{sim}(g_{i},g_{j})$.

The contrastive loss is defined as


(10) $$L_{\text{ctr}}=\sum_{i\in P}\left[-\frac{1}{\left|P(i)\right|}\sum_{p\in P(i)}\;\text{log}\;\frac{\;\text{exp}\;\left(\text{sim}(g_{i},g_{p})/\tau \right)}{\sum_{a\in (P\cup U)\setminus \{i\}}\;\text{exp}\;\left(\text{sim}(g_{i},g_{a})/\tau \right)}\right],$$

where τ>0 is a temperature hyperparameter controlling the sharpness of the similarity distribution.

This loss pulls embeddings of labeled protective antigens closer together, while simultaneously pushing them away from unlabeled proteins, which may include non-protective or ambiguous cases. By operating at the graph level, the contrastive objective complements the task loss by improving global representation geometry and promoting robust separation across diverse proteomes.

## 4 Results

In this section, we present the results of PU-GRAIL across three protective antigen prediction benchmark datasets: Vaxign-ML, ImmunoDB, PAPreC. We first assess model performance on established benchmark datasets using standard evaluation metrics, including area under the precision–recall curve (AUPR), area under the receiver operating characteristic curve (AUROC), F1 score, and Matthews correlation coefficient (MCC).

### 4.1 PAPreC dataset

We first evaluated PU-GRAIL on the PAPreC benchmark, which includes antigenicity prediction tasks spanning multiple sequence lengths and biological contexts, namely epitope-level (Bcipep and HlA) and protein-level datasets. All evaluations were conducted using 5-fold cross-validation under identical training and evaluation splits to ensure a fair comparison across methods. As summarized in Table 2, PU-GRAIL showed consistent improvements across all four datasets, demonstrating its ability to generalize across both short epitope sequences and long protein sequences. Notably, the performance advantage of PU-GRAIL was most pronounced on the short-sequence epitope datasets (Bcipep and HlA), where existing methods exhibited substantially reduced performance or, in some cases, were unable to generate predictions. In particular, Vaxign-ML and VenusVaccine, which are primarily designed for protein-level antigenicity prediction, could not be reliably applied to short sequence inputs.

**Table 2:** Performance comparison on the PAPreC benchmark.^a^

Model	Epitope		Protein		Bcipep		HLA	
	AUPR	AUROC	AUPR	AUROC	AUPR	AUROC	AUPR	AUROC
VaxiJen 2.0	0.52±0.01	0.52±0.02	0.54±0.02***	0.59±0.02	0.83±0.01	0.54±0.05	0.13±0.01	0.46±0.02
VaxiJen 3.0	0.57±0.02	0.51±0.02	0.65±0.02***	0.58±0.01	0.85±0.03	0.52±0.06	0.22±0.01*	0.45±0.01
Vaxign-ML^b^			0.94±0.01***	0.92±0.01				
PAPreC	0.69±0.00***	0.78±0.01	0.92±0.02***	0.92±0.00	0.92±0.01***	0.71±0.03	0.37±0.05***	0.71±0.02
VenusVaccine^b^			**0.96** ± **0.01** ***	0.95±0.01	0.89±0.02***	0.71±0.02	0.32±0.04*	0.70±0.02
PU-GRAIL	**0.86** ± **0.01** ***	**0.86** ± **0.01**	**0.96** ± **0.00** ***	**0.96** ± **0.00**	**0.96** ± **0.01** ***	**0.82** ± **0.04**	**0.44** ± **0.04** ***	**0.75** ± **0.03**

a AUPR *P*-values were computed using the Mann–Whitney *U*-test: *P<0.05, **P<0.01, ***P<0.001. Confirmed. All figures and text are original or appropriately cited. Bold values indicate the best-performing method for each metric.

b Methods that do not support prediction on short peptide sequences are indicated by “–”.

### 4.2 Vaxign-ML dataset

To further assess the generalization ability of PU-GRAIL across distinct bacterial lineages, we conducted an external evaluation using the Gram-positive and Gram-negative test sets provided by the Vaxign-ML benchmark. These test sets consist of experimentally validated protective antigens and non-antigens curated independently from PAPreC, enabling a strictly independent assessment without data leakage.

PU-GRAIL was trained on the protein-level dataset from PAPreC using 5-fold cross-validation. For each fold, all sequences overlapping with the Vaxign-ML benchmark were removed from the corresponding training split to ensure strict independence between training and test data. The trained models from each fold were then directly evaluated on the Gram-positive and Gram-negative test sets from Vaxign-ML without any further fine-tuning, and performance was reported as the mean and standard deviation across the five folds.

In Table 3, PU-GRAIL achieved competitive performance on both Gram-positive and Gram-negative test sets, demonstrating its ability to generalize beyond the bacterial species and data distributions observed during training. These results indicate that PU-GRAIL captures transferable antigenicity patterns rather than overfitting to dataset-specific characteristics of PAPreC. For comparison, results for VaxiJen 2.0 and VaxiJen 3.0 are reported as single-point estimates obtained from their respective web servers, as these methods do not support retraining or cross-validation on user-defined datasets. Consequently, their reported performance does not reflect 5-fold cross-validation but rather a single evaluation on the corresponding test sets.

**Table 3:** Performance comparison on the Vaxign-ML benchmark.^a^

Model	Protegen Gram−				Protegen Gram+			
	AUPR	AUROC	F1	MCC	AUPR	AUROC	F1	MCC
VaxiJen 2.0^b^	0.62***	0.69	0.66	0.19	0.63	0.60	0.74	0.22
VaxiJen 3.0^b^	0.73***	0.69	0.68	0.67	0.80***	0.72	0.71	0.33
Vaxign-ML	0.69±0.04***	0.72±0.03	0.67±0.01	**0.72** ± **0.03**	0.81±0.03***	0.78±0.02	0.76±0.01	0.21±0.07
PAPreC	0.68±0.05***	0.67±0.05	**0.72** ± **0.02**	0.35±0.14	0.79±0.08***	0.71±0.05	0.71±0.05	**0.46** ± **0.06**
VenusVaccine	0.30±0.02	0.66±0.02	0.69±0.09	0.27±0.21	0.82±0.04**	0.70±0.02	0.76±0.04	0.28±0.22
**PU-GRAIL**	**0.82** ± **0.10** ***	**0.78** ± **0.05**	0.71±0.05	0.71±0.10	**0.84** ± **0.08** ***	**0.79** ± **0.12**	**0.78** ± **0.06**	0.34±0.25

a AUPR *P*-values were computed using the Mann–Whitney *U*-test: *P<0.05, **P<0.01, ***P<0.001. Bold values indicate the best-performing result for each metric.

b VaxiJen 2.0 and VaxiJen 3.0 are web server-based methods with fixed pretrained models; as retraining is not possible, predictions are deterministic and standard deviations across folds are not reported.

Overall, this external validation highlights the robustness of PU-GRAIL under realistic vaccine discovery settings, where models are required to prioritize antigen candidates for previously unseen bacterial strains.

### 4.3 ImmunoDB dataset

To further assess the versatility of PU-GRAIL across heterogeneous antigen types, we evaluated its performance on the ImmunoDB benchmark, which integrates antigenicity prediction tasks spanning bacterial, viral, and tumor vaccine antigens. In contrast to PAPreC and Vaxign-ML, which are primarily designed for bacterial antigen discovery, ImmunoDB offers a unified evaluation framework encompassing both pathogen-derived and self-associated antigen candidates. This design enables a systematic comparison of model performance across biologically distinct sequence classes within a single benchmark. In Table 4, PU-GRAIL consistently demonstrated strong performance across all three ImmunoDB subsets. On the bacterial and viral datasets, PU-GRAIL outperformed or matched existing methods in terms of AUPR, AUROC, and F1 score, indicating robust generalization across diverse microbial proteomes. These results suggest that PU-GRAIL captures antigenicity-related patterns that are shared across pathogen types, rather than relying on organism-specific or dataset-specific biases. Notably, PU-GRAIL also achieved competitive and stable performance on the binary tumor vaccine dataset, which represents a particularly challenging setting due to the self-derived nature of tumor-associated antigens and the lack of a clear non-self signal. In this context, several baseline methods exhibited unstable or inflated performance, likely reflecting reliance on coarse physicochemical heuristics. In contrast, PU-GRAIL maintained balanced discrimination, suggesting that it learns transferable antigenic representations that extend beyond simple compositional features. We did not include VaxiJen 3.0 in the ImmunoDB-tumor comparison because its training data overlap with the ImmunoDB-tumor test set. Taken together, these results demonstrate that PU-GRAIL generalizes effectively across a broad spectrum of antigenic sequences, ranging from pathogen-derived proteins to self-associated tumor vaccine candidates. This consistent performance across heterogeneous antigen classes highlights the suitability of PU-GRAIL as a general-purpose antigenicity prediction framework, rather than a model tailored to a single biological domain. To further examine the generalization capability of PU-GRAIL across different antigen classes, we conducted an additional cross-dataset evaluation. Specifically, models trained on one ImmunoDB subset (virus, bacteria, or tumor) were directly applied to the other subsets without retraining. The results, summarized in Fig. 1, available as supplementary data at *Bioinformatics* online, show that PU-GRAIL maintains stable predictive performance across cross-species settings. In particular, models trained on pathogen-derived datasets (virus or bacteria) still achieve reasonable AUPR and AUROC when evaluated on tumor-associated antigens, despite the substantial biological differences between these antigen classes. These results suggest that PU-GRAIL learns transferable antigenicity-related representations that generalize beyond dataset-specific characteristics.

**Table 4:** Performance comparison on the ImmunoDB benchmark.^a^

Model	ImmunoDB-tumor			ImmunoDB-bacteria			ImmunoDB-virus		
	AUPR	AUROC	F1	AUPR	AUROC	F1	AUPR	AUROC	F1
VaxiJen 2.0^b^	0.44±0.01	0.57±0.02	0.48±0.02	0.69±0.05***	0.81±0.02	0.70±0.01	0.73±0.02***	0.80±0.01	0.83±0.01
VaxiJen 3.0^b^				0.82±0.02 ***	0.84±0.01	0.76±0.02	0.84±0.01***	0.77±0.02	0.76±0.01
Vaxign-ML	0.56±0.07***	0.71±0.04	0.58±0.05	0.80±0.03***	0.86±0.01	0.75±0.02	0.96±0.01***	0.96±0.01	0.90±0.01
PAPreC	0.69±0.01***	0.79±0.03	0.67±0.02	0.78±0.02***	0.83±0.01	0.88±0.02	0.95±0.01***	0.94±0.01	0.89±0.01
VenusVaccine	0.73±0.06***	0.84±0.03	0.74±0.05	0.80±0.06***	0.87±0.02	0.75±0.03	0.96±0.01***	0.95±0.01	0.88±0.07
PU-GRAIL	**0.81** ± **0.04** ***	**0.87** ± **0.03**	**0.77** ± **0.01**	**0.83** ± **0.02** ***	**0.89** ± **0.02**	**0.77** ± **0.03**	**0.97** ± **0.00** ***	**0.97** ± **0.01**	**0.92** ± **0.00**

a AUPR was assessed by the Mann–Whitney *U*-test: *P<0.05, **P<0.01, ***P<0.001. Bold values indicate the best-performing result for each metric.

b VaxiJen 2.0 and VaxiJen 3.0 are web-server-based methods; the model is fixed across folds and only the test sets differ.

### 4.4 Ablation study

To quantify the contribution of each learning objective in PU-GRAIL, we performed an ablation study by systematically removing individual loss components from the full training objective. Here, we denote the PU-AUC task loss as *T*, the node-level separation loss as *N*, and the graph-level contrastive loss as *C*. We compared the full model trained with all losses (T+N+C) against variants that drop the node-level separation loss (T+C), drop the graph-level contrastive loss (T+N), or use the task objective alone (*T*). We additionally report partial baselines that exclude the task loss (N+C, *N* only, *C* only) to illustrate the necessity of PU-aware task supervision.

**Figure 2:**
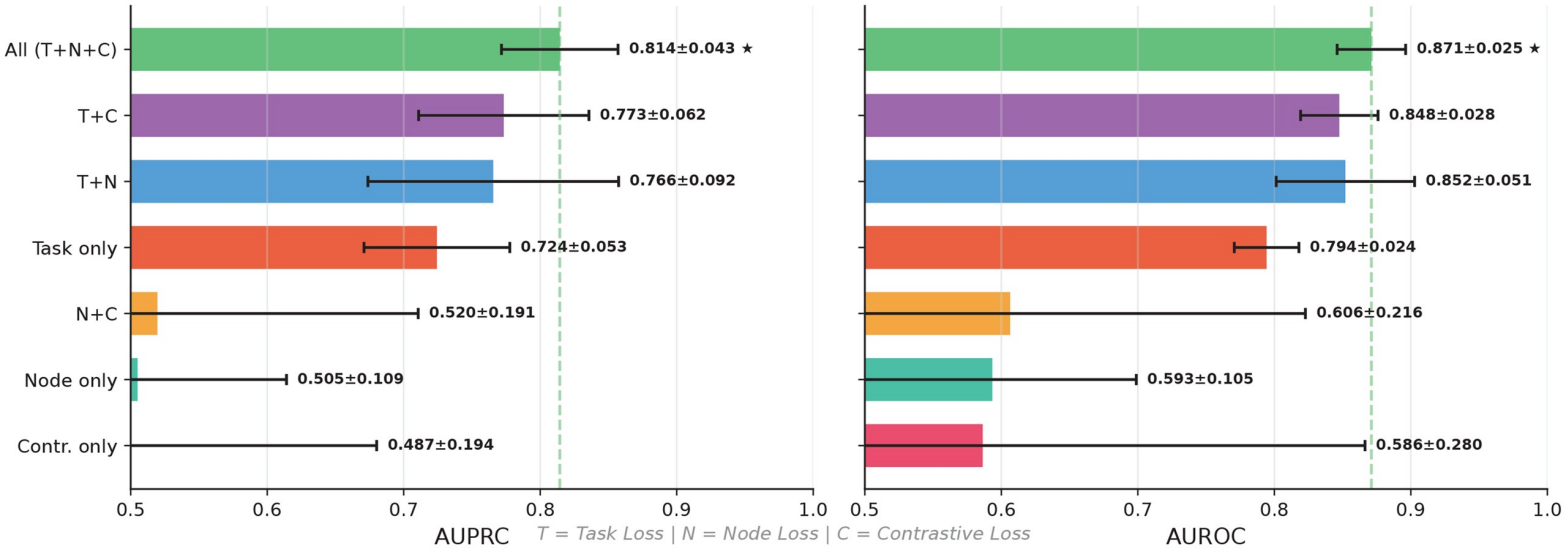
AUPR (left) and AUROC (right) of PU-GRAIL variants trained with different combinations of the three objectives: task loss (*T*), node-level separation loss (*N*), and graph-level contrastive loss (*C*) in ImmunoDB tumor sequence. Bars show mean ± SD across runs under identical data splits and hyperparameters; the dashed line indicates the full model.

**Figure 3:**
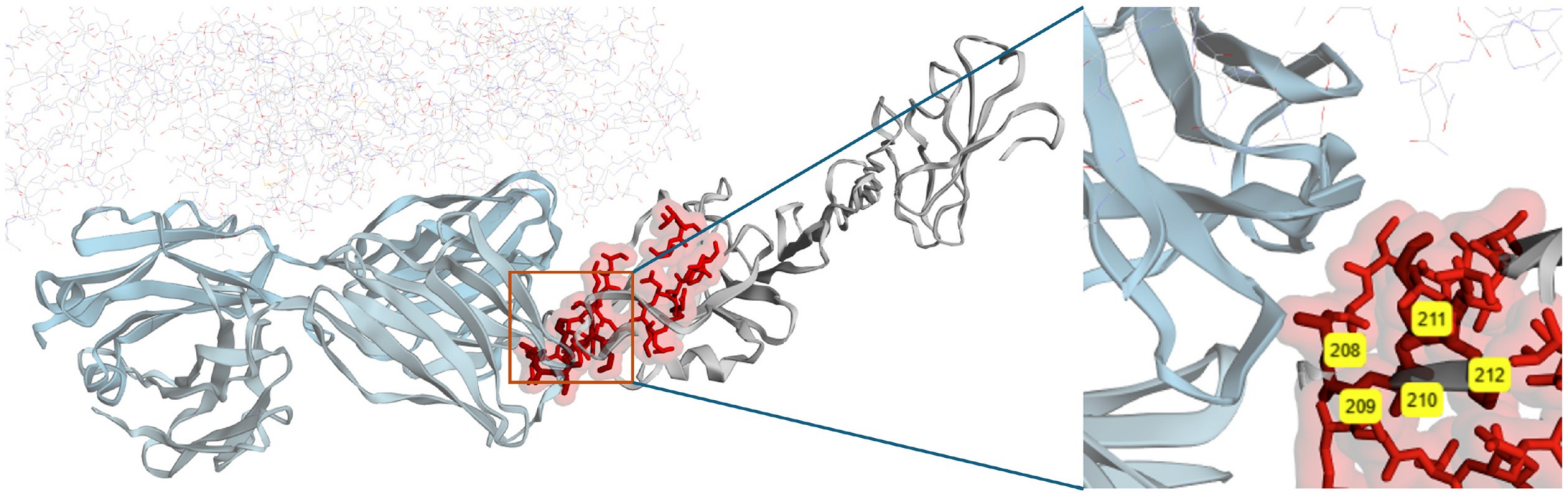
Structural mapping of attention scores onto bacterial antigen–antibody complexes. Representative visualization of the 1FJ1 complex showing the bacterial antigen bound to antibody fragments. Residues are annotated by attention score: the top 10% residues are highlighted, with the top 10 shown with stronger emphasis; the top five residues are labeled by residue number (e.g. 208, 209, 211, 212).

**Figure 4:**
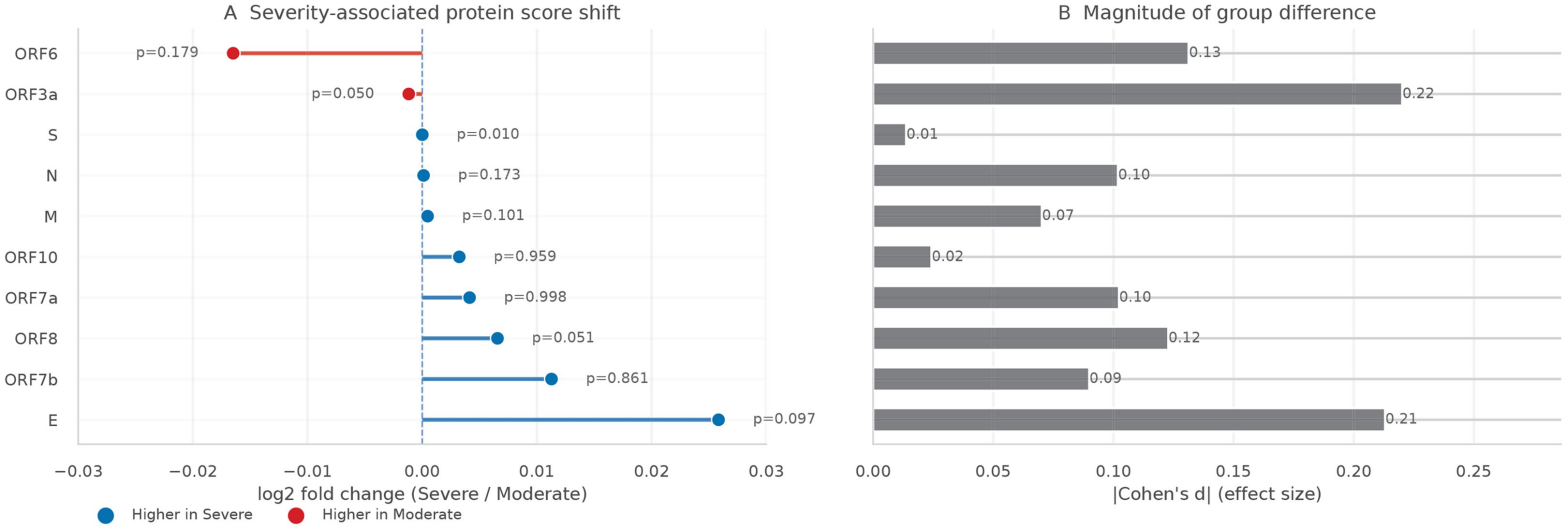
(A) Protein-level antigenicity score differences between severe and moderate COVID-19 patient groups, quantified as log_2_ fold change (severe/moderate). Positive values indicate higher predicted antigenicity in severe cases, whereas negative values indicate higher scores in moderate cases. Adjusted *q*-values from statistical testing are shown for each protein. (B) Magnitude of group differences measured by the absolute value of Cohen’s *d*, reflecting effect size independent of direction. Accessory proteins such as ORF3a and ORF7b exhibit larger severity-associated shifts compared with structural proteins.

All variants were trained and evaluated under identical data splits and hyperparameter settings. Performance was assessed using AUPR and AUROC, which are appropriate for the highly imbalanced antigenicity prediction setting. We report results on the ImmunoDB Tumor benchmark in Fig. 2, and provide corresponding ablations on bacterial and viral datasets in Figs 2 and 3, available as supplementary data at *Bioinformatics* online, respectively. Overall, the full objective (T+N+C) achieved the best performance, indicating that the node-level and graph-level regularizers provide complementary benefits beyond the task loss alone. Removing either *N* or *C* led to consistent performance drops, while models trained without the task loss (N+C, *N* only, *C* only) performed substantially worse, confirming that PU-aware task supervision is essential and that the auxiliary objectives are most effective when coupled with the primary antigenicity prediction loss. We further provide a consolidated comparison of PU-GRAIL variants evaluating edge design (sequence, structural, and dynamic kNN) and structure predictor (AlphaFold2, OmegaFold, ESMFold) in Figs 4 and 5, available as supplementary data at *Bioinformatics* online.

### 4.5 Bacterial vaccine antigen discovery

To identify candidate vaccine antigens from bacterial proteomes, we evaluate whether predicted candidates preferentially recover experimentally validated protective antigens across multiple bacterial species. Following established practice in reverse vaccinology, we assess whether candidate prioritization effectively enriches known immunogens. For fair comparison with existing approaches, we adopt the evaluation protocol (Dalsass *et al.* 2019), which benchmarks models across 11 bacterial strains with experimentally validated protective antigens. Accordingly, we compared our approach with VenusVaccine, PAPreC, and Vaxign-ML across the same set of 11 bacterial species using the benchmark dataset described in Table 2, available as supplementary data at *Bioinformatics* online.

To compare the ability of different methods to prioritize protective antigens, we evaluated candidate rankings using three complementary criteria: fold enrichment, recall, and score fold change (SFC). Proteins were ranked in descending order of predicted scores, and the top 30 candidates were selected. Fold enrichment at K=30 was computed as


$$\text{FE}@30=\frac{\left(\frac{P_{30}}{30}\right)}{\left(\frac{P}{N}\right)},$$

where $P_{30}$ denotes the number of known bacterial protective antigens among the top 30 ranked proteins, *P* is the total number of known protective antigens, and *N* is the total number of proteins in the corresponding bacterial proteome. This metric quantifies the degree to which known antigens are overrepresented among highly ranked candidates relative to their background frequency. In addition, we evaluated recall, defined as the proportion of known protective antigens recovered within the top-ranked set, which measures the sensitivity of each method in identifying experimentally validated antigens. Finally, score fold change (SFC) was used to assess the separation between predicted scores of protective antigens and non-antigen proteins, with larger values indicating stronger discriminative power. The results for fold enrichment, recall, and SFC are summarized in Tables 3–5, available as supplementary data at *Bioinformatics* online. Together, these metrics provide complementary perspectives on candidate prioritization, antigen recovery, and score discrimination, enabling a comprehensive comparison across the 11 bacterial species.

To validate the biological relevance of our attention mechanism, we examined whether high-attention residues correspond to antibody-binding sites in experimentally resolved structures. We selected two bacterial antigen-antibody complexes from the Protein Data Bank: 1FJ1, the *Staphylococcus aureus* enterotoxin B (SEB) in complex with a neutralizing antibody, and 2YPV, the *Bordetella pertussis* pertactin autotransporter bound to its cognate antibody.

For each complex, we computed residue-level attention scores and calculated the minimum distance from each antigen residue to any antibody atom. As shown in Fig. 3 and Fig. 6, available as supplementary data at *Bioinformatics* online, high-attention residues (top 10% by score) were predominantly located within 4.5 Å of antibody atoms, corresponding to direct epitope-paratope contact distances. This structural correspondence demonstrates that PU-GRAIL identifies immunologically relevant surface regions without explicit structural supervision.

### 4.6 Severity-associated antigenicity analysis in SARS-CoV-2

We next examined whether PU-GRAIL captures clinically relevant severity-associated immune signals in real-world SARS-CoV-2 infection. To this end, we analyzed an independent cohort of 348 COVID-19 patients collected by the Korea National Institute of Health (KNIH; Jo *et al.* 2022), including 302 patients classified as moderate (WHO ordinal scale < 7) and 46 classified as severe (WHO ordinal scale ≥ 7). For each patient, amino acid sequences of SARS-CoV-2 proteins corresponding to immunologically relevant genes were obtained, and overlapping 12-mer epitope windows were generated for each protein.

At the protein level, predicted antigenicity scores were compared between moderate and severe patient groups for each gene. Figure 4 shows severity-associated antigenicity score shifts across SARS-CoV-2 proteins in severe and moderate COVID-19 patient groups. Panel A displays log2 fold changes in predicted antigenicity scores between severe and moderate groups, with adjusted q-values shown for each protein. Panel B displays effect sizes measured by the absolute value of Cohen’s d. ORF3a and Spike show statistically significant score shifts, while E protein and ORF3a show the largest effect sizes.To further characterize severity-associated patterns at higher resolution, we conducted an epitope-level analysis. For each epitope, patient-level mean scores were compared between groups using a two-sided Mann-Whitney *U* test. Epitope-level significance was defined using a nominal threshold of p<0.05. Gene-level summaries were computed as the fraction of significant epitopes relative to the total number of testable epitopes for each gene. This epitope-centric analysis revealed substantial heterogeneity across the SARS-CoV-2 proteome. We summarize gene-level heterogeneity as the protein-wise fraction of severity-discriminative epitopes (Fig. 7, available as supplementary data at *Bioinformatics* online). ORF3a and Spike protein exhibited the highest fractions of severity-discriminative epitopes, whereas structural proteins such as N and M showed comparatively lower proportions. Both ORF3a and E proteins have been experimentally validated as SARS-CoV-2 viroporins capable of activating inflammasome pathways. However, ORF3a has been identified as a particularly potent inducer of macrophage inflammasome activation and pulmonary IL-1α release, whereas E protein elicits more context dependent and heterogeneous inflammatory responses (Geanes *et al.* 2024). These mechanistic differences provide a biological rationale for the more consistent severity-associated antigenicity patterns observed for ORF3a compared to E protein in our analysis.

In contrast, the Spike (S) protein, while not a viroporin, plays a central role in viral entry and is the primary target of host adaptive immunity. Spike-derived epitopes have been shown to be broadly and consistently recognized across patients, leading to robust and conserved immune responses (Harvey *et al.* 2021). Nevertheless, recent studies have reported that immune responses to Spike can vary substantially depending on mutations at specific hotspot regions that are associated with disease severity (Youn *et al.* 2025). Consistent with these observations, the Spike protein exhibited statistically significant severity-associated shifts at both the protein and epitope levels with PU-GRAIL.

## 5 Discussion

Overall, PU-GRAIL provides a structure-aware framework for protective antigen prediction under a positive-unlabeled learning setting. By integrating protein sequence embeddings with predicted 3D structures in a residue-level graph representation, the model captures both local sequential context and long-range spatial contacts relevant to epitope recognition. The use of a PU-AUC objective addresses inherent label uncertainty in antigen datasets, as supported by ablation analyses. Attention-based pooling enables residue-level interpretation, with high-attention residues aligning with antibody–antigen interfaces in experimentally resolved complexes. Beyond benchmark datasets, the framework identified severity-associated antigenicity differences in SARS-CoV-2 proteins, including ORF3a and Spike. Limitations include reliance on predicted structures, which may be less reliable for disordered regions. Future work may incorporate structural confidence measures and extend the approach to broader pathogen classes such as parasitic or fungal antigens.

## Author Contribution

Jaemin Jeon (Data curation [lead], Formal analysis [lead], Investigation [equal], Methodology [lead], Resources [equal], Software [lead], Validation [lead], Visualization [lead]), Sangwook Jung (Data curation [supporting], Formal analysis [supporting], Validataion [supporting]), Inuk Jung (Data curation [supporting], Formal analysis [supporting]), Kwangsoo Kim (Conceptualization [lead], Investigation[lead], Supervision [lead], Funding acquisition [lead], Project administration [lead]), and Jinki Yeom (Conceptualization [lead], Investigation [lead], Supervision [lead], Project administration [lead])

## Supplementary Material

btag263_Supplementary_Data

## Data Availability

The datasets of the COVID-19 cohort used in this study are available online in the Clinical and Omics Data Archive (CODA) database All benchmark datasets used for protective antigen prediction are publicly available and were obtained from the resources provided by the respective publications: **Vaxign-ML**: the dataset and the independent test split released by Ong *et al*. (2020). **ImmunoDB**: the publicly available dataset released by Li *et al*. (2024). **PAPreC**: the publicly available benchmark dataset released by Martins *et al*. (2025).
