# Supplementary material for: PU-GRAIL: residue-level graph learning for identifying protective bacterial antigens under positive-unlabeled supervision
Source: Bioinformatics. 2026 Jul 7;42(Suppl 1):btag263. doi: 10.1093/bioinformatics/btag263 (PMC13341136; doi:10.1093/bioinformatics/btag263)

Supplementary Table S1: Train–test overlap validation across datasets. For each dataset, we report the mean sequence identity and mean structural similarity between training and test samples, averaged over all sampled train–test pairs and across five folds. Sequence identity is computed via global alignment (0–1 scale), and structural similarity is measured using TM-score (0–1 scale), where values below 0.5 indicate dissimilar folds. Across all datasets, both sequence identity and structural similarity remain low, confirming minimal homology and no evidence of sequence or structural leakage between training and test folds.

Dataset	Sequence mean similarity	Structure mean similarity
ImmunoDB-tumor	0.1198	0.221
ImmunoDB-virus	0.3041	0.262
ImmunoDB-bacteria	0.0908	0.244
Bcipep	0.2821	0.274
All Gram epitope	0.2861	0.328
All Gram protein	0.2724	0.269
Hla	0.1928	0.223

Supplementary Table S2: Summary of the 100 experimentally validated bacterial protective antigens used in this study. The table covers 11 bacterial species, reporting for each species the total number of protein sequences analyzed and the number of known bacterial protective antigens.

Pathogen	Strain	Toal number of Sequences	Number of Known Bacterial Antigens
Neisseria gonorrhoeae	ATCC700825	2,106	10
Borrelia burgdorferi	ATCC35210	1,294	6
Campylobacter jejuni	ATCC700819	902	7
Chlamydia pneumoniae	ATCC VR-2282	1,623	7
Escherichia coli	CFT073	5,343	10
Treponema pallidum	Nichols	569	11
Streptococcus pneumoniae	TIGR4	2,002	4
Neisseria gonorrhoeae	ATCC700825/ FA 1090	2,114	14
Helicobacter pylori	J99	1,038	4
Streptococcus pyogenes	M1	1,693	15
Staphylococcus Aureus	MW2	915	12

Supplementary Table S3: Comparison of fold enrichment at K = 30 across four antigen prediction models. Fold enrichment was computed as the enrichment of known bacterial protective antigens among the top 30 ranked proteins relative to their background frequency in the full dataset.

Top30	**PU-Grail**	**VenusVaccine**	**PAPreC**	**Vaxign-ML**
Neisseria gonorrhoeae	28.1	21.1	21.0	**35.1**
Borrelia burgdorferi	**28.1**	**28.1**	14.2	21.4
Campylobacter jejuni	**28.1**	12.9	12.9	7.5
Chlamydia pneumoniae	**28.3**	23.2	23.2	23.2
Escherichia coli	**17.6**	**17.6**	**17.6**	**17.6**
Treponema pallidum	**23.2**	8.6	13.8	9.5
Streptococcus pneumoniae	**70.3**	49.9	33.3	33.3
Neisseria gonorrhoeae	**15.1**	10.0	15.1	10.0
Helicobacter pylori	**16.9**	8.4	0.0	**16.9**
Streptococcus pyogenes	**24.7**	17.6	24.7	17.6
Staphylococcus Aureus	7.62	7.62	**10.1**	**10.1**

Supplementary Table S4: Comparison of recall across four antigen prediction models for 11 bacterial species. Recall represents the proportion of known bacterial protective antigens successfully recovered among the top-ranked candidate proteins for each species.

Recall	PU-Grail	VenusVaccine	PAPreC	Vaxign-ML
Neisseria gonorrhoeae	0.9	0.8	1.0	0.9
Borrelia burgdorferi	1.0	0.7	1.0	1
Campylobacter jejuni	0.8	0.43	0.71	0.71
Chlamydia pneumoniae	1.0	1.0	1.0	1.0
Escherichia coli	0.86	0.9	1.0	1.0
Treponema pallidum	1.0	0.5	1.0	1.0
Streptococcus pneumoniae	1.0	1.0	1.0	0.93
Neisseria gonorrhoeae	1.0	0.80	0.86	1.0
Helicobacter pylori	1.0	0.25	1.0	1.0
Streptococcus pyogenes	1.0	0.88	1.0	1.0
Staphylococcus Aureus	1.0	0.83	1.0	1.0

Supplementary Table S5: Comparison of score fold change (SFC) across four antigen prediction models for 11 bacterial species. Score fold change (SFC) quantifies the relative separation between the predicted scores of known bacterial protective antigens and non-antigen proteins, with higher values indicating improved discrimination.

**Score Fold chang (SFC)**	**PU-Grail**	**VenusVaccine**	**PAPreC**	**Vaxign-ML**
Neisseria gonorrhoeae	**3.7**	2.8	2.6	1.4
Borrelia burgdorferi	**4.0**	3.04	2.4	1.47
Campylobacter jejuni	**2.2**	1.7	2.0	1.35
Chlamydia pneumoniae	**7.0**	4.3	2.8	1.42
Escherichia coli	**3.8**	3.0	2.8	1.58
Treponema pallidum	**9.1**	3.8	2.9	1.40
Streptococcus pneumoniae	**4.1**	3.1	2.9	1.57
Neisseria gonorrhoeae	**2.5**	2.4	2.2	1.33
Helicobacter pylori	**2.5**	2.0	2.0	1.51
Streptococcus pyogenes	**3.9**	2.3	2.6	1.42
Staphylococcus Aureus	**4.2**	2.4	2.6	1.38

Supplementary Figure S1: Ablation study on ImmunoDB bacteria benchmarks. AUPRC (left) and AUROC (right) for PU-GRAIL ablations across bacterial datasets, comparing different combinations of task loss (T), node-level loss (N), and contrastive loss (C). Bars report mean ± s.d. across runs under matched splits and hyperparameters.

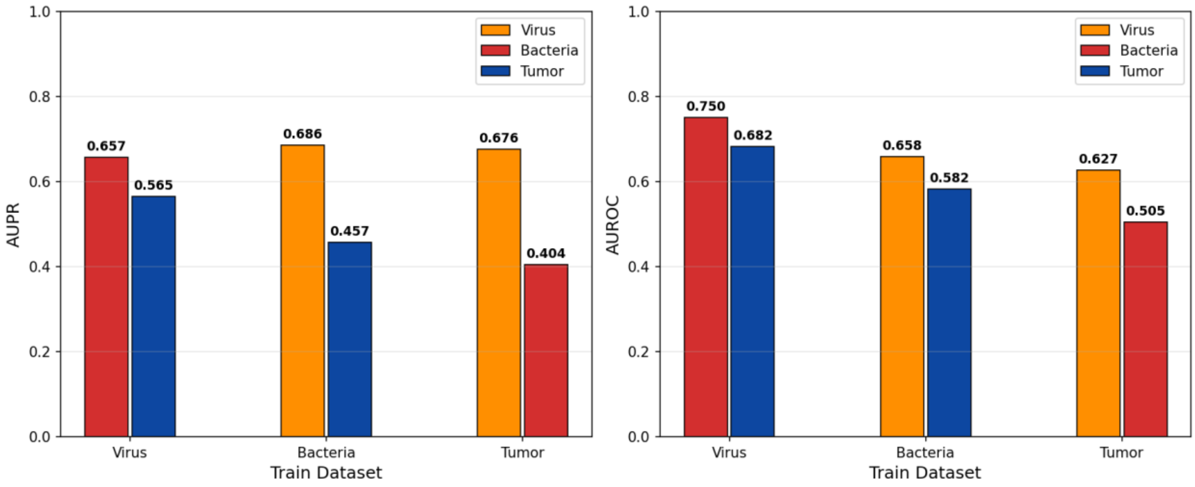

Supplementary Figure S2: Ablation study on ImmunoDB bacteria benchmarks. AUPRC (left) and AUROC (right) for PU-GRAIL ablations across bacterial datasets, comparing different combinations of task loss (T), node-level loss (N), and contrastive loss (C). Bars report mean ± s.d. across runs under matched splits and hyperparameters.

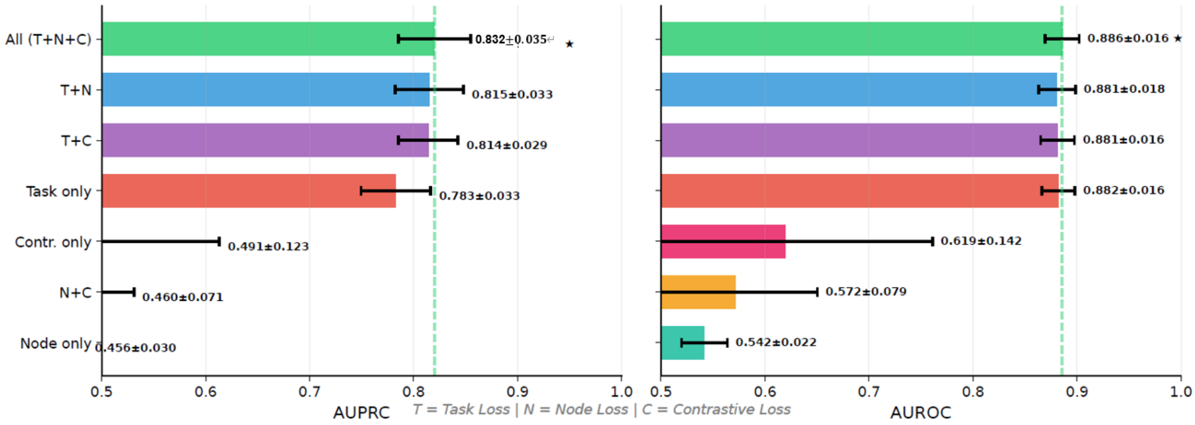

Supplementary Figure S3: AUPRC (left) and AUROC (right) for PU-GRAIL variants on viral datasets, evaluating the contribution of task loss (T), node-level separation (N), and graph-level contrastive learning (C). Bars report mean ± s.d. across runs under identical evaluation settings.

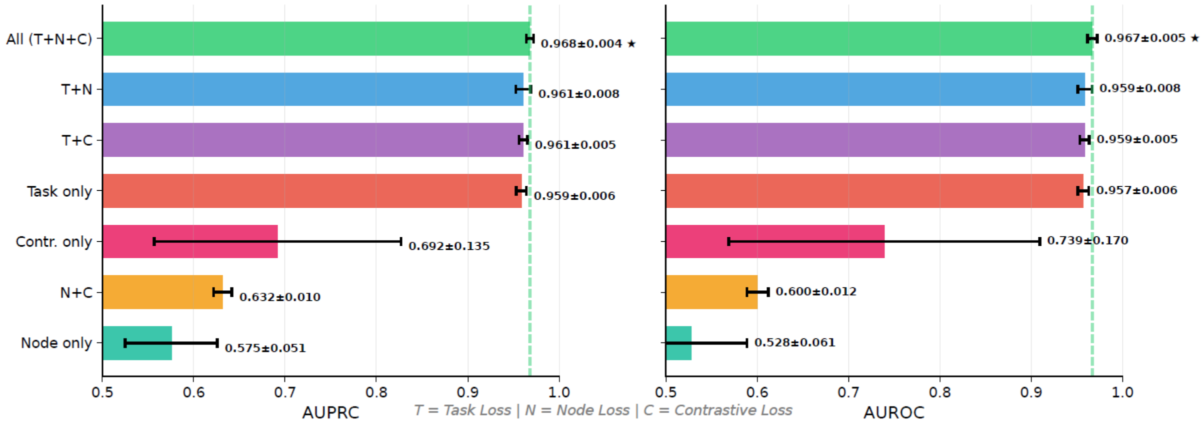

Supplementary Figure S4: AUPRC (left) and AUROC (right) for PU-GRAIL variants on viral datasets, evaluating the contribution of task loss (T), node-level separation (N), and graph-level contrastive learning (C). Bars report mean ± s.d. across runs under identical evaluation settings.

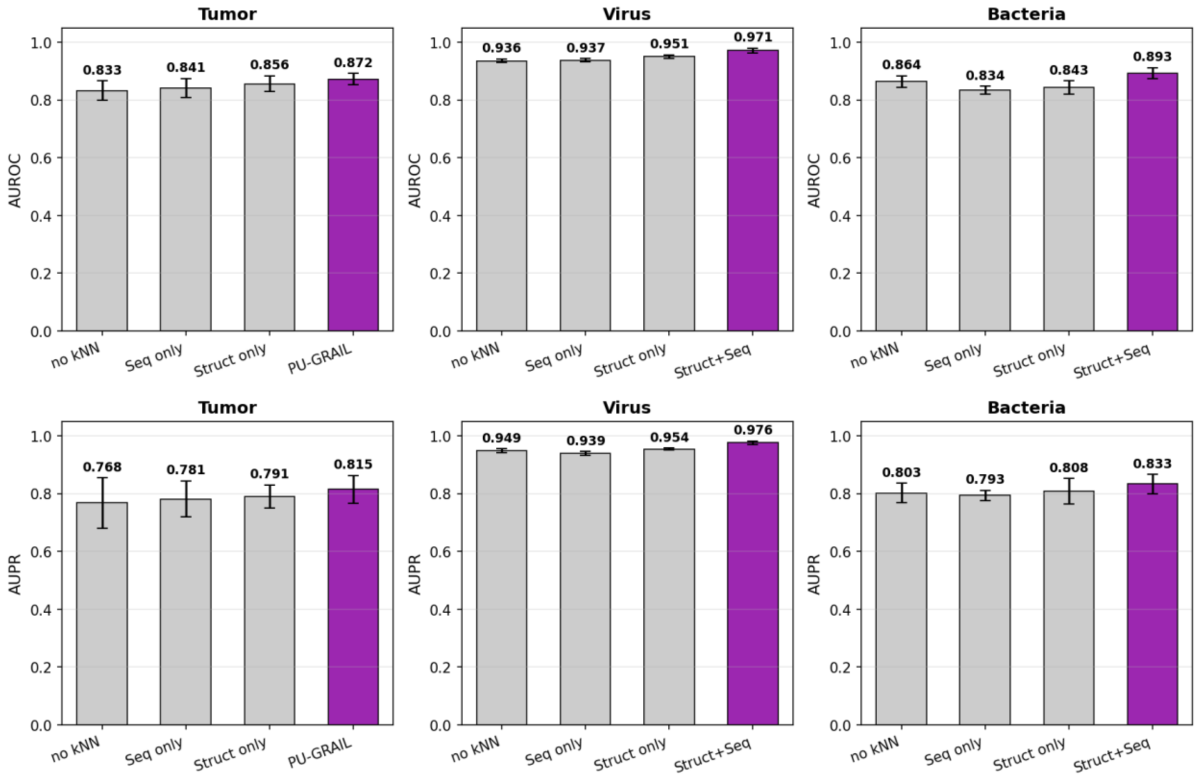

Supplementary Figure S5: Comparison of AUPR across structure predictors with mean structural confidence (pLDDT) overlay. **AUPR performance of the proposed model using structures predicted by AlphaFold2, ESMFold, and OmegaFold across three datasets (Tumor, Virus, and Bacteria). Bars represent mean AUPR values, and error bars indicate standard deviation across runs. The black line denotes the mean pLDDT score of predicted structures for each dataset, reflecting structural confidence.**

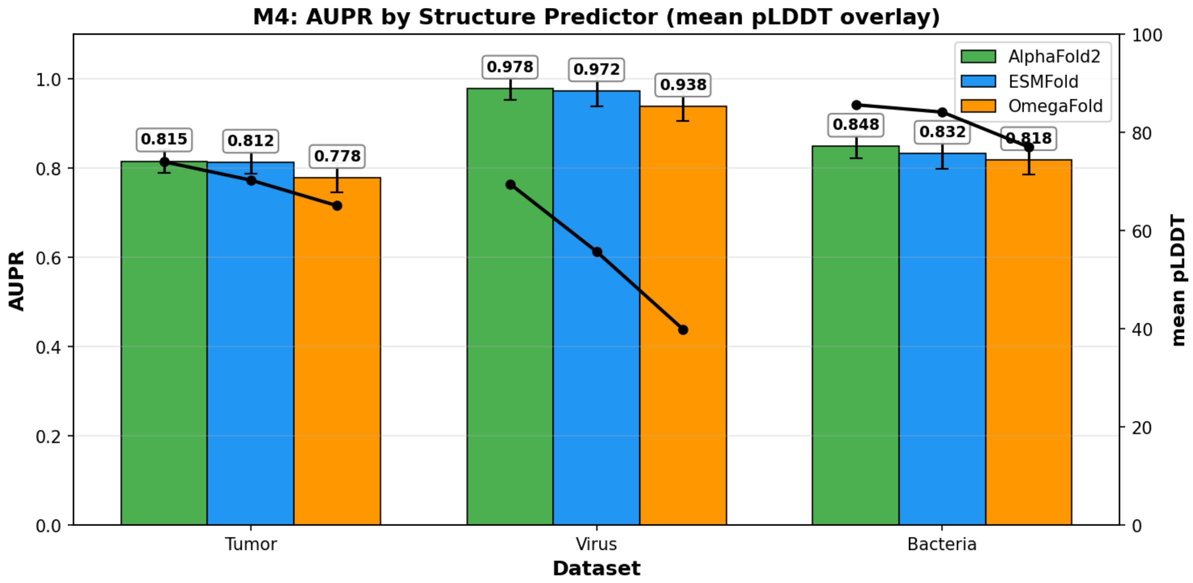

Supplementary Figure S6: Attention-based epitope prediction on the 2YPV antibody-antigen complex. Structural visualization showing the bacterial antigen (light blue ribbon) in complex with antibody fragments (gray ribbon). High-attention residues identified by our model are displayed in red stick representation with corresponding residue numbers labeled (210, 214, 216, 217, 219). Consistent with the 1FJ1 analysis, high-attention residues localize precisely at the antibody-antigen binding interface, further validating the model's ability to identify epitope regions.

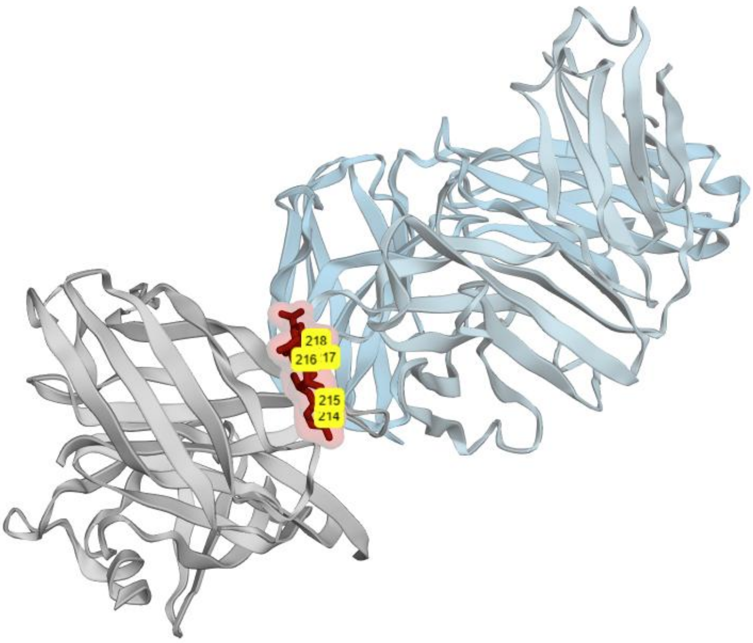

Supplementary Figure S7: Protein-wise distribution of significant epitopes.
Fraction of statistically significant epitope windows across SARS-CoV-2 proteins (10 genes), highlighting proteins enriched for discriminative epitope signals.

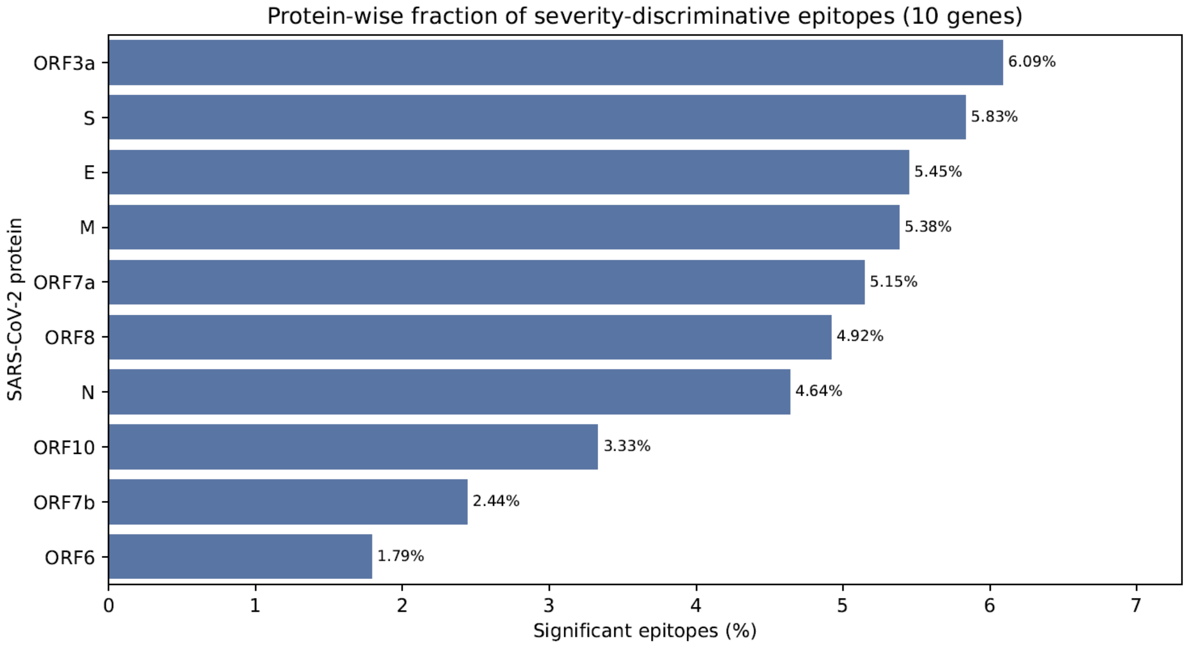

Supplement: btag263_Supplementary_Data [file btag263_supplementary_data.pdf]
